# Deep sequencing of New World screw-worm transcripts to discover genes involved in insecticide resistance

**DOI:** 10.1186/1471-2164-11-695

**Published:** 2010-12-08

**Authors:** Renato A Carvalho, Ana Maria L Azeredo-Espin, Tatiana T Torres

**Affiliations:** 1 Centro de Biologia Molecular e Engenharia Genética (CBMEG), Universidade Estadual de Campinas (UNICAMP), Campinas, SP, Brazil; 2 Departamento de Genética e Evolução, Universidade Estadual de Campinas (UNICAMP), Campinas, SP, Brazil; 3 Departamento de Genética e Biologia Evolutiva, Instituto de Biociências, Universidade de São Paulo (USP), São Paulo, SP, Brazil

## Abstract

**Background:**

The New World screw-worm (NWS), *Cochliomyia hominivorax*, is one of the most important myiasis-causing flies, causing severe losses to the livestock industry. In its current geographical distribution, this species has been controlled by the application of insecticides, mainly organophosphate (OP) compounds, but a number of lineages have been identified that are resistant to such chemicals. Despite its economic importance, only limited genetic information is available for the NWS. Here, as a part of an effort to characterize the *C. hominivorax *genome and identify putative genes involved in insecticide resistance, we sampled its transcriptome by deep sequencing of polyadenylated transcripts using the 454 sequencing technology.

**Results:**

Deep sequencing on the 454 platform of three normalized libraries (larval, adult male and adult female) generated a total of 548,940 reads. Eighteen candidate genes coding for three metabolic detoxification enzyme families, cytochrome P450 monooxygenases, glutathione S-transferases and carboxyl/cholinesterases were selected and gene expression levels were measured using quantitative real-time polymerase chain reaction (qRT-PCR). Of the investigated candidates, only one gene was expressed differently between control and resistant larvae with, at least, a 10-fold down-regulation in the resistant larvae. The presence of mutations in the acetylcholinesterase (target site) and carboxylesterase E3 genes was investigated and all of the resistant flies presented E3 mutations previously associated with insecticide resistance.

**Conclusions:**

Here, we provided the largest database of NWS expressed sequence tags that is an important resource, not only for further studies on the molecular basis of the OP resistance in NWS fly, but also for functional and comparative studies among Calliphoridae flies. Among our candidates, only one gene was found differentially expressed in resistant individuals, and its role on insecticide resistance should be further investigated. Furthermore, the absence of mutations in the OP target site and the high frequency of mutant carboxylesterase E3 indicate that metabolic resistance mechanisms have evolved predominantly in this species.

## Background

Until very recently, the database of genomic sequences for insect species has been restricted to model species. Now, with the recent advances in DNA sequencing technology, the generation of sequence data has increased at an unprecedented rate. As new sequencing technologies became less expensive, it is possible to generate genomic information from non-model species. This holds a great promise for several species with medical, veterinary or economic importance.

New World screw-worm (NWS), *Cochliomyia hominivorax*, is one of the most important insect pests in South and Central America [1]. NWS myiasis is caused by the larval stage of the fly infesting tissues of warm-blooded vertebrates. Such infestations cause significant losses in the livestock industry through morbidity, mortality and the cost of treating infested animals. This insect pest also represents a serious public health problem in the Caribbean region, where screw-worm infestations in humans are frequently reported [2]. Historically, NWS was widely distributed from the southern U.S. to central Argentina. However, this species has been successfully eradicated from North and most of Central America by the Sterile Insect Technique [3]. In South America and in the Caribbean region, however, this pest continues to affect the development of the livestock sector and wider economic development.

In its current geographical distribution, NWS has been controlled exclusively by chemical insecticides, in particular organophosphate (OP) and pyrethroid-based compounds [4]. Nevertheless, the intensive use of these chemicals has led to the selection of resistant strains, which, in turn, compromises the effective control of NWS. In this context, the elucidation of the molecular basis of insecticide resistance in NWS is of great value to minimize its effect by adopting resistance management strategies. The major mechanisms of insecticide resistance already described in several insects involve the alteration of target sites inducing insensitivity to the insecticide (target-site resistance) and/or an increase in the rate of insecticide metabolism (metabolic resistance) [5]. The metabolic resistance may result from coding sequence alteration of metabolic genes and/or over-expression of enzymes capable of metabolizing the insecticide [6].

Despite its medical and veterinary importance and its negative economic impact on the livestock sector, only limited genetic information is available for the NWS. Molecular studies in this species focused on the characterization of molecular markers in the mitochondrial [7] and nuclear genomes [8,9], their utilization in population genetic studies [10-13] and the characterization of genes and substitutions involved in insecticide resistance [4,14,15]. In a recent study, one substitution was found in the acetylcholinesterase gene (AChE), whose product is the target of OP, but with a very low frequency in several NWS populations [16]. No substitutions were found in the sodium channel gene, target of pyrethroids [15]. In contrast, substitutions in the carboxylesterase E3 gene with very high frequencies were found in several NWS populations surveyed [14-16]. This gene has been previously associated with OP resistance in the sheep blowfly *Lucilia cuprina *and in the housefly *Musca domestica *[17,18]. In this enzyme, a G137D substitution in the oxyanion hole within the active site results in diethyl OP resistance and a second substitution, W251L, in the acyl pocket of the active site also confers resistance to OPs, mainly dimethyl OP compounds, and pyrethroids [19,20]. However, there is no information currently available on metabolic resistance in NWS based on up-regulation of detoxification enzymes such as cytochrome P450 monooxygenases, glutathione S-transferases and carboxyl/cholinesterases.

Now, the combined availability of a rapidly growing database of insect genomic sequences and the recent developments in sequencing technology provides an opportunity for genome-wide gene discovery in *C. hominivorax*, including genes that might be involved in insecticide resistance. Parallel sequencing of short cDNA fragments has been demonstrated as an excellent tool to generate genome-wide sequence information [21-24] as well as levels of gene expression [25,26].

Here, as a part of an effort to characterize the *C. hominivorax *genome and identify putative genes involved in insecticide resistance, we sampled its transcriptome by deep sequencing of polyadenylated transcripts using the 454 sequencing technology. In this study, we report the analysis of ~500,000 expressed sequence tags (ESTs) generated from three libraries (adult male, adult female and larvae), providing, to our knowledge, the largest EST database for a Calliphoridae species. We anticipate that the availability of these sequences will be of significant value to functional studies in *C. hominivorax *and closely related species in the Calliphoridae family.

Furthermore, we performed larval bioassays to select individuals resistant to an OP insecticide. The presence of acetylcholinesterase (target site) and carboxylesterase E3 mutations was investigated and quantitative polymerase chain reaction following reverse transcription (qRT-PCR) was used to identify differentially expressed genes putatively involved in metabolic resistance. Our results add to the basic knowledge of the molecular mechanisms involved in OP resistance and are of potential applied interest to assist the design of new and more effective strategies for controlling NWS.

## Results

### Assembly

**Figure 1:**
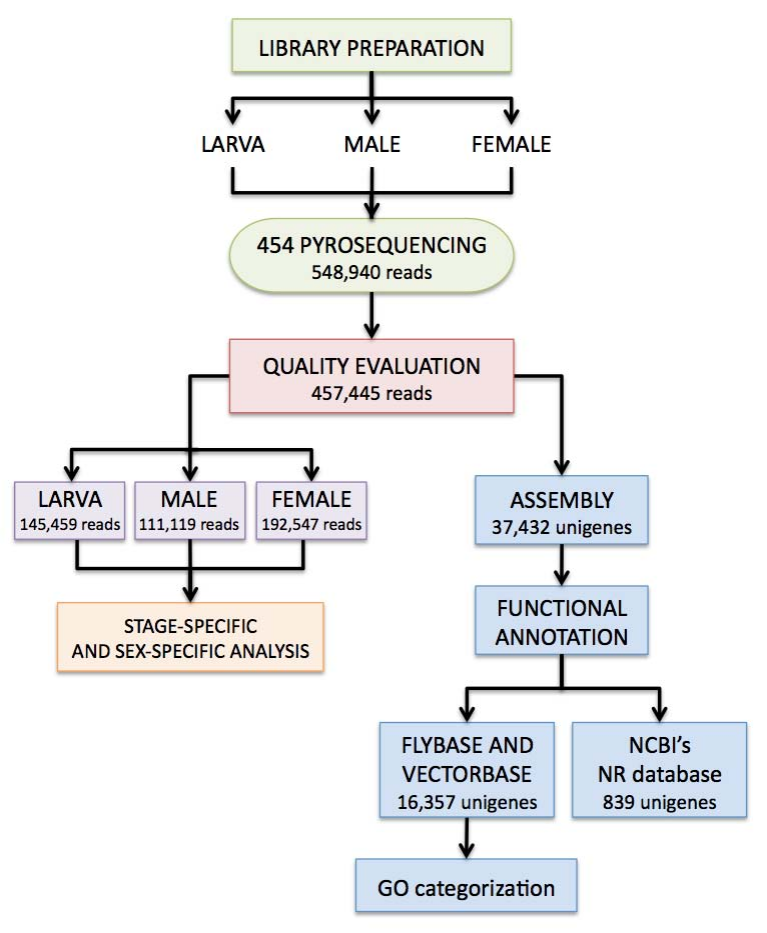
**Pipeline for the generation and analysis of *C. hominivorax *ESTs**.

Deep sequencing on the 454 platform of the three normalized libraries generated a total of 548,940 reads of raw nucleotide sequence data with an average read length of 184 bp after the removal of low score ends (Table 1). An overview of the pipeline for the analysis of *C. hominivorax *ESTs is presented in Figure 1.

**Table 1:** Summary of C. hominivorax 454-EST data.

MID	Sample	Number of reads	High quality reads*	Average read length
CGTGTCTCTA	Larvae	174,459	145,964	187 pb
CTCGCGTGTC	Male adults	132,646	111,119	184 pb
TAGTATCAGC	Female adults	232,332	192,547	182 pb
Not found	Not atributed	9,503	7,815	171 pb

Total	All	548,940	457,445	184 pb

Reads from male, female and larval libraries were sorted by their unique multiplex identifiers sequences (MID)

After quality evaluation, 457,445 of the obtained reads were assembled using MIRA [27]. A subset of these reads. 357,355 (78%), was considered in the assembly, which resulted in 37,432 unigenes (36,650 contigs and 782 singlets). The mean unigene size was 315 bp, with an average coverage of 5.2 sequences per nucleotide position. The average contig size obtained is consistent with previous reports describing EST sequencing using the 454 technology for different insect species, *Melitaea cinxia *[24], *Sarcophaga crassipalpis *[22] and *Chrysomela tremulae *[21].

**Figure 2:**
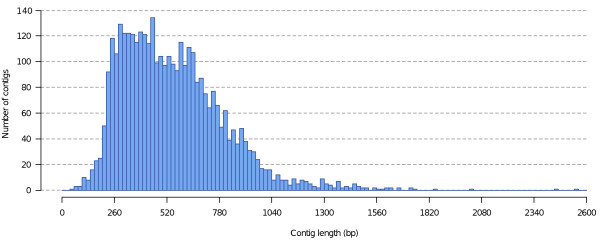
**Distribution of contig length**. Histogram based on a subset of contigs formed by the assembly of at least 20 reads (3,630 contigs), the average contig size increased to 550 bp.

The average length of unigenes is affected by the high number of singletons and contigs formed by the assembly of very few short reads. This drawback resulting from the use of NGS is ameliorated by the depth of coverage. Increased sequence depth results in increase length coverage of cDNA sequence. Taking a subset of contigs formed by the assembly of at least 20 reads (3,630 contigs), the average contig size increased to 550 bp (Figure 2). Sampling the 10% longest contigs (3,743 contigs), the average contig size increased to 660 bp with an average coverage of 9.8 sequences per nucleotide position. This sub-sampling showed that we have more than 3,700 contigs with very good coverage, quality and length for downstream analysis.

### Functional annotation of *C. hominivorax *unigenes

**Figure 3:**
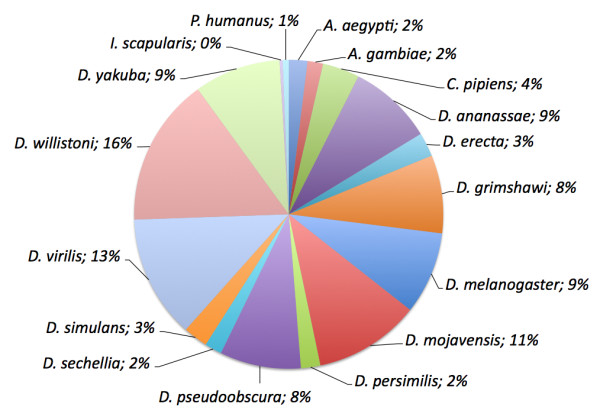
**Percentage of *C. hominivorax *unigenes mapped to the different databases used for automated annotation**. tBLASTx searches were performed for each unigene against the last releases of annotated transcripts from the twelve Drosophila genomes (Flybase) and *Aedes aegypti*, *Anopheles gambiae*, *Culex pipiens*, *Ixodes scapularis *and *Pediculus humanus *(Vectorbase). Approximately 44% of the NWS unigenes were mapped against this database.

To infer the functional identity of the NWS unigenes, we mapped each unigene sequence to annotated transcripts from *Anopheles gambiae, Aedes aegypti, Culex pipiens, Ixodes scapularis*, *Pediculus humanus *and the 12 sequenced *Drosophila *species (Additional file 1) using tBLASTx [28] with relaxed criteria (e < 10^-4^, > 50% identity, > 20% of read length included in the high scoring segment pair, HSP). About 44% (16,357) of the NWS unigenes could be mapped to this database of protein-coding transcripts from the 17 insect species (Figure 3 Additional file 1). Each NWS unigene was automatically assigned a gene annotation corresponding to the hit in the insect database with the highest E-value and that hit was considered as a putative homolog of the NWS gene. The number of NWS unigenes mapped to each insect library reflects the size of the library as well as the divergence of NWS to each insect species.

The remaining unigenes (20,990) were remotely BLASTed against NCBI's non-redundant database and 839 had an acceptable hit against this library under our criteria (e < 10^-4^, > 50% identity, > 20% of read length included in the HSP). Blasting *C. hominivorax *unigenes against the NCBI's 'nr' database, we obtained 180 hits to bacterial sequences that are either in association with *C. hominivorax *or formerly present in the blood and/or meat used in the rearing media (data available upon request)

**Figure 4:**
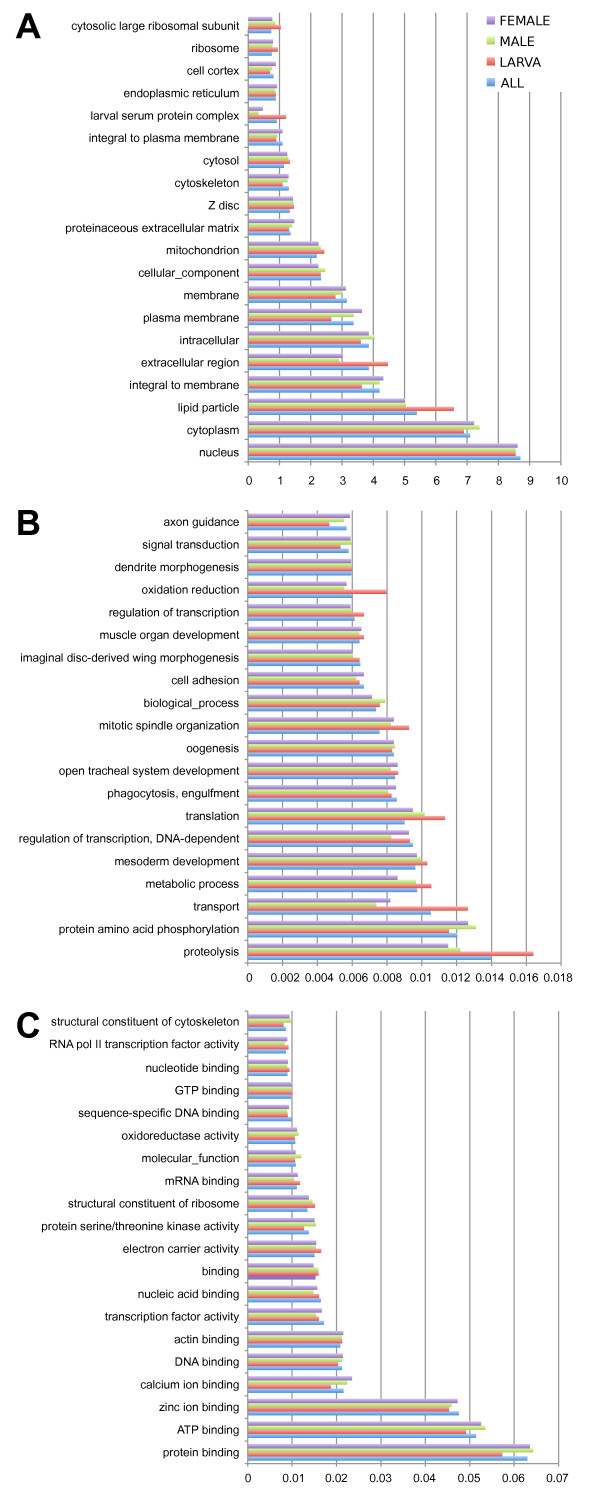
**Top 20 Gene Ontology classes in the three *C. hominivorax *libraries**. A comparison of the distribution across mainly represented Gene Ontology (GO) classes in our three libraries and in the pooled dataset. Twenty categories are shown for each (A) Biological process, (B) Molecular function and (C) Cellular component.

Gene ontology (GO) classifications [29] of the corresponding *D. melanogaster *orthologs were obtained from Flybase [30]. NWS unigenes were classified into the three GO categories: biological process, molecular function, and cellular component, according to the standard GO terms [31]. The top 20 most represented GO categories in the pooled dataset are illustrated in Figure 4. Under the category of biological process, proteolysis, protein amino acid phosphorylation, transport, mesoderm development and regulation of transcription were among the most highly represented categories, reflecting the most important metabolic activities in *C. hominivorax*. The representation of the GO categories were very similar between male and female libraries. In larval libraries, some categories were over-represented, reflecting fundamental processes enriched in larvae, such as proteolysis, transport and the accumulation of lipids.

Some genes belonging to GO classes putatively involved in organophosphate resistance were selected for gene expression evaluation: cholinesterase, carboxylesterase, juvenile-hormone esterase, serine-type carboxypeptidase, glutathione transferase and monooxygenase activities (Table 2).

**Table 2:** Candidate genes selected for gene expression analysis via qRT-PCR

Gene family	Gene name	*D. melanogaster *ID	Number of unigenes	Number of reads
*Esterases*	Acethylcholinesterase	FBgn0000024	3	13
	Alpha-Esterase-7 *EST7*	FBgn0015575	1	49
	Alpha-Esterase-8 *EST8*	FBgn0015576	3	15
	Alpha-Esterase-9 *EST9*	FBgn0015577	1	20
	Juvenilehormone esterase	FBgn0010052	3	28
	Serineprotease 7	FBgn0037515	4	42

*GST*	GlutathioneS transferase D1	FBgn0001149	14	205
	GlutathioneS transferase E5	FBgn0063495	3	194
	GlutathioneS transferase S1	FBgn0010226	5	223

*P450*	Cyp4ac1	FBgn0031693	2	8
	Cyp4c3	FBgn0015032	1	2
	Cyp4d2	FBgn0011576	3	12
	Cyp6a14	FBgn0033302	5	46
	Cyp6a9	FBgn0013771	13	132
	Cyp6d4	FBgn0039006	4	30
	Cyp6g1	FBgn0025454	4	14
	Cyp6v1	FBgn0031126	2	14
	Cyp9f2	FBgn0038037	2	4
	Cyp12a4	FBgn0038681	8	75

The number of unigenes represents the number of assembled contigs that were mapped to the corresponding gene in D. melanogaster and the number of reads represents the the sum of the number of reads that were used for the assembly of each contig.

### Analysis of sex-specific and stage-specific transcripts

**Figure 5:**
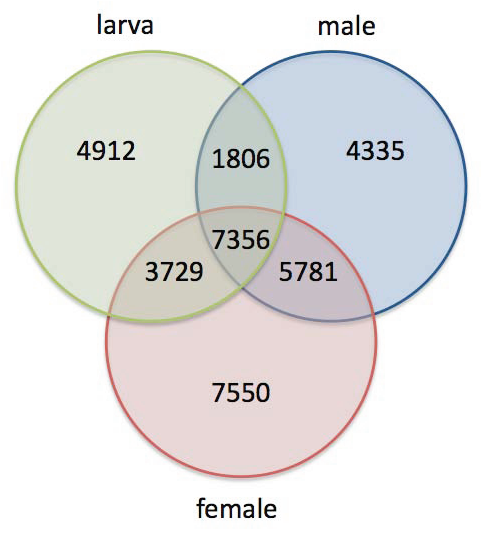
**Venn diagram showing the distribution of reads in the three *C. hominivorax *libraries**. Reads from each library were mapped against NWS unigenes. This allowed the identification of reads shared between the three libraries, reads shared between two of them and library-specific reads.

**Figure 6:**
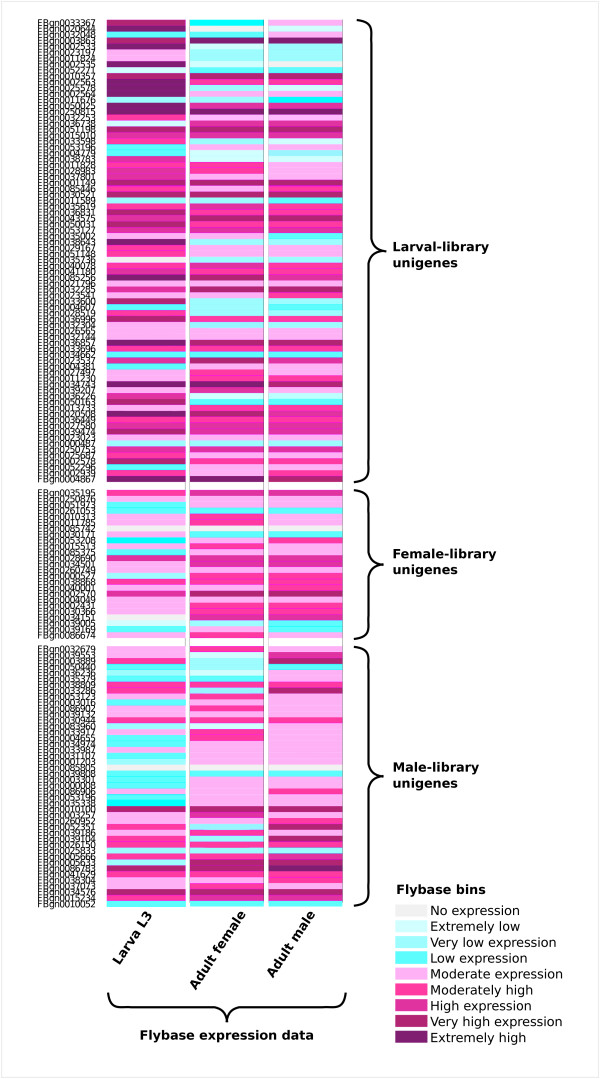
**Analysis of the gene expression levels of the *D. melanogaster *orthologs of the NWS library-specific unigenes**. For each library-specific unigene we recorded the gene level high throughput expression data of the putative ortholog in *D. melanogaster *obtained from Flybase. The expression levels are sorted in bins that are represented by different colours. We used the same bins and colours as in Flybase. The *D. melanogaster *orthologs are displayed from the highest number of reads in the NWS libraries (top) to the lowest (bottom) in each library. The NWS larval-specific unigenes also showed a higher expression level in the putative ortholog in *D. melanogaster*. We only included in this analysis NWS unigenes represented by at least 10 reads in the male library, which corresponded to 13 and 17 reads in the larval and female library, respectively.

Reads from male, female and larval libraries were sorted by their unique multiplex identifiers sequences (MID) and the number of 454-ESTs derived from each library is presented in Table 1. To identify sex-and stage-specific transcripts, we mapped the sorted reads against NWS unigenes (i.e., we performed similarity searches against the database of NWS unigenes). The largest library, adult female, showed the highest number of exclusive (library-specific) unigenes (Figure 5), due to a higher chance of sampling rare transcripts. To reduce this artefact, we normalized the read counts by the total number of reads in each library and chose a threshold of a minimum representation (in number of reads) for any given unigene. The threshold was set to reduce the number of shared unigenes sampled by chance in only one library. We chose an arbitrary threshold of 5 reads in the smallest library (male), which corresponded to 7 reads in the larval library and 9 reads in the female library. In fact, the female library had the smallest number of exclusive unigenes with a sufficient representation (289 unigenes). The larval library had the highest number of exclusive unigenes (628), closely followed by the male library (586 exclusive unigenes). Approximately 40% of the female-and male-specific unigenes were mapped to the database of *D. melanogaster *transcripts as described above (121 and 236 unigenes, respectively). Almost 55% of the larval-specific transcripts were mapped to the same database (339 unigenes). For each library-specific unigene we recorded the gene-level high-throughput expression data of the putative ortholog in *D. melanogaster *obtained from Flybase [30]. We used the expression data from L3 Larva (Puff stage 1-2), 1-day-old adult males and 1-day-old adult females (Figure 6). By using this approach we could assess if the library-specific genes indeed have a stage-or sex-biased expression. We could only find a consistent pattern in the larval-specific genes; the unigenes that were found exclusively in our larval library also showed a higher expression level of the putative ortholog in *D. melanogaster*. Among the putative orthologs with a clear larval-biased expression in *D. melanogaster *were four larval cuticle proteins, two larval serum proteins and three cuticular proteins, known for their higher expression in larvae. As expected, for most male- and female-exclusive unigenes we could not recover a sex-biased expression in *D. melanogaster *orthologs. Sex-biased genes often evolve more rapidly, both in coding and regulatory sequence [reviewed in [32]]. Hence, one would not expect the conservation of expression levels between these two highly divergent species.

In non-normalized cDNA samples, the number of reads mapping to a specific unigene should be proportional to its level of expression. However, differences in transcript abundance in our normalized libraries may reflect different normalization efficiency rather than genuine differences in gene expression. Nevertheless, highly abundant library-specific transcripts are potential candidates for differentially expressed genes among the different libraries. Hence, we performed an analysis to identify such genes by aligning the sorted reads against all unigenes. The number of reads from each library normalized by the library size was used as a measure of the transcript abundance. Comparing the larval library and the combined adult libraries, we found 645 unigenes with at least a 10-fold difference in the representation of reads (Additional file 2). In our comparison between sexes (Additional file 3), the number of transcripts with different representation was 101 (with at least a 10 fold difference). The top 20 unigenes with a different representation had an average fold change of 29 in the comparison between larval and adult samples and of 46 in the comparison between male and female library. Most likely, the fold changes reported here are underestimations of the different representation of the transcripts in the three libraries, due to the small dynamic range of this analysis, but our purpose was to indicate some candidate genes for further studies, rather than a genome-wide gene expression profiling.

### Comparison to publicly-available *C. hominivorax *sequences

We compared our ESTs to NWS sequences available in public databases. Most of these sequences were non-annotated ESTs sequenced using the Sanger technology from a single study [33]. We assembled the 18,000 publicly-available ESTs (Acession numbers FG282693-FG301340) into 3,439 contigs and 2,694 singlets and mapped our unigenes against this database using BLAST [34]. The comparison between our library and the Sanger sequences showed that only 6,719 of our unigenes have a hit in the Sanger library. The high number of non-mapped hits in our library against the former one reflects the recovery of low expressed transcripts due to the higher throughput of our analysis.

To determine the source of the non-mapped transcripts, we aligned all 454-reads sorted to each library (larval, adult males, adult females) against the assembled Sanger sequences using PanGEA [35]. Of the total good quality reads in each library, we could map 31,950 (22%), 32,088 (17%) and 16,864 (15%) to the larval, adult female and adult male libraries, respectively. As expected, the largest number of mapped reads originates from the larval library, given that the Sanger libraries were derived from *C. hominivorax *larvae and eggs.

### Analysis of genes related to secondary metabolism

Genes coding for three enzyme families, cytochrome P450 monooxygenases (*CYP*), glutathione S-transferases (*GST*) and carboxyl/cholinesterases (*CCE*), putatively involved in metabolic detoxification of insecticides were selected after the functional annotation against *D. melanogaster *database [30]. NWS unigenes mapped to 34 *D. melanogaster *genes of the *CYP *family. Of these, 28 corresponded to *CYP *subfamilies found to be involved in insecticide resistance in previous studies [reviewed in [36]] and among them, 15 belonged to the *CYP6*, eight to the *CYP4 *and five to the *CYP12 *subfamilies. Seven *CCE *and six *GST *genes were also found among NWS unigenes. All NWS unigenes that mapped to these *D. melanogaster *genes were remotely searched in NCBI (blastx) to confirm the identity of the NWS sequences. Candidate genes (Table 2) for the gene expression analysis using quantitative real time PCR (qRT-PCR) were selected based on unambiguous alignments between NWS unigenes and *D. melanogaster *annotated genes.

### Carboxylesterase and acetylcholinesterase genotyping

Bioassays with the dimethyl organophosphate dichlorvos (dimethyl 2,2-dichlorovinyl phosphate) performed in duplicate with 500 NWS larvae selected 44 and 58 resistant individuals in each replicate (R1 and R2, respectively). A non-treated group was used as a control for further analyses. To identify allelic variants possibly involved in OP resistance, we genotyped three substitutions (I298V, G401A e F466Y) in the AChE gene previously associated with OP resistance in *D. melanogaster *and *L. cuprina *[37,38]. These three substitutions occur at key sites located within the active site gorge of this enzyme. The alteration of these amino acids results in a reduced sensitivity to OP and carbamate insecticides [37,38].

**Figure 7:**
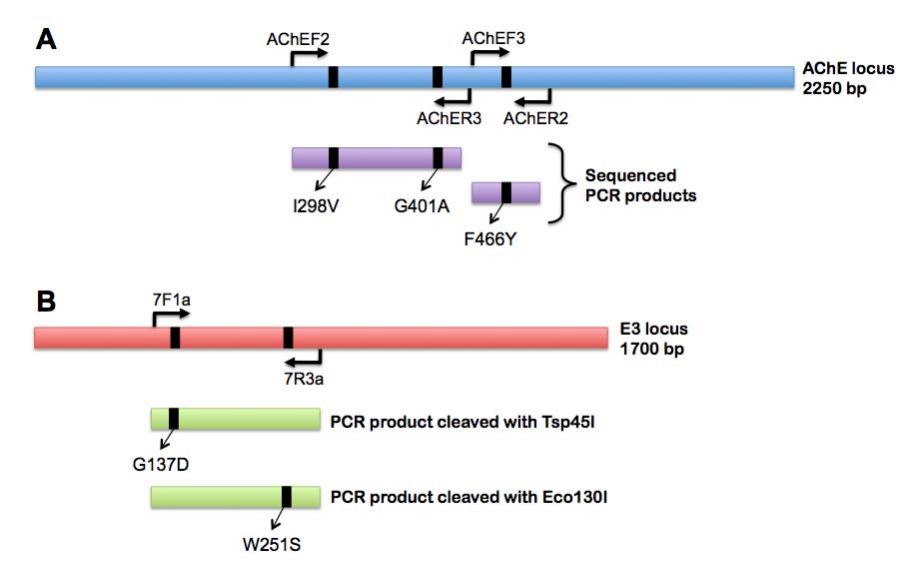
**Carboxylesterase and acetylcholinesterase genotyping**. Schematic representation of the loci and methods for the genotyping of the acetylcholinesterase (A) and carboxylesterase (B) substitutions.

Two regions containing these three substitutions (Figure 7A) were amplified from the cDNA synthesized using the RNA pools from each group, control (C) and resistant group 1 (replicate 1, R1). The PCR products were directly sequenced and only the wildtype allele was observed.

Polymerase Chain Reaction-Restriction Fragment Length Polymorphism (PCR-RFLP) was used to identify two substitutions, G137D and W251S, previously characterized in NWS carboxylesterase gene, E3 [4,14] and associated with insecticide resistance (Figure 7B). The G137D substitution in the oxyanion hole within the active site of the enzyme causes a shift in the enzyme function, from a carboxylesterase to an OP hydrolase activity. This shift confers resistance to OP insecticides with a preference for diethyl OPs. The second substitution, W251S, in the acyl pocket of the active site also confers resistance to OP insecticides, but with this substitution, the enzyme acquires a higher affinity for dimethyl OP compounds [18]. Of 40 individuals analyzed from the control group, 17 showed a substitution only on the first site (G137D), 13 only on the second site (W251S) and 10 on both sites. Individuals with both mutations had been cloned and sequenced previously [4] showing only one mutation in each allele, according to studies in *L. cuprina *[39]. However, all 44 resistant individuals (R1 group) were homozygous for the mutated allele, with the W251S substitution, indicating a strong association between this mutation and dichlorvos (a dimethyl OP) resistance.

### Quantitative measure of gene expression in OP resistant larvae

**Figure 8:**
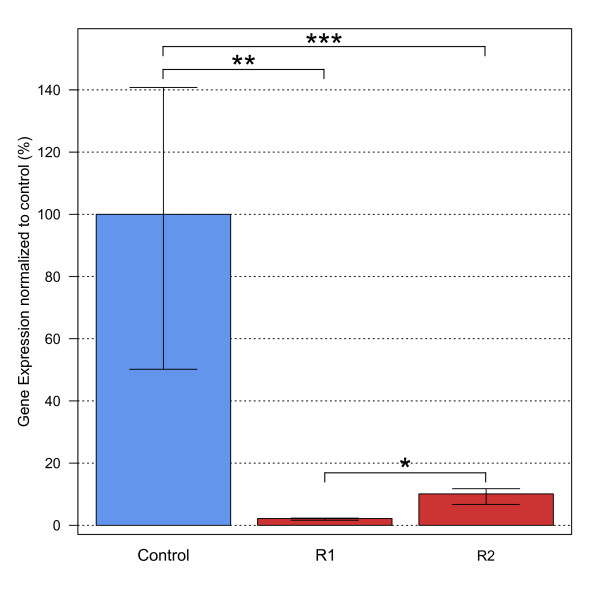
**Expression of *cyp6g1 *in control and resistant samples**. The gene expression level of *cyp6g1 *was measured for the control and resistant samples by qRT-PCR In both resistant groups, R1 and R2, a the gene was down-regulated when compared to the control group. It was approximately 45-fold down-regulated in R1 and 10-fold in R2. *, p < 0.01; **, p < 0.001; ***, p < 0.0001

Gene expression levels were measured for 18 candidate genes by qRT-PCR (Table 2 Additional file 4). Of these, only one showed significant differences between control and resistant groups 1 (R1) and 2 (R2). In both resistant groups, R1 and R2, a putative ortholog to the *cyp6g1 *gene was down-regulated when compared to the control group. A one-way ANOVA between control and resistant groups was conducted to compare the levels of gene expression of *cyp6g1*. There was a significant difference on gene expression levels [F(2, 6) = 83.61, p = 0.00004]. Post hoc comparisons using the Tukey HSD test indicated that the expression levels of *cyp6g1 *were significantly reduced in both resistant samples, R1 (p = 0.00003) and R2 (p = 0.0006). This gene was approximately 45-fold and 10-fold down-regulated in R1 in R2, respectively (Figure 8).

## Discussion

Given the economic importance of *C. hominivorax *it is surprising that only now there is an interest in functional studies involving this livestock pest. Here, we took advantage of NGS to sequence and characterize *C. hominivorax *expressed sequence tags from three normalized libraries prepared with larval, adult female and adult male samples. We used the different normalized libraries to sample stage- and sex-specific transcripts involved in NWS metabolism, and therefore, candidates for an involvement in metabolic insecticide resistance. Furthermore, we wanted to generate a database of stage-specific transcripts that can be useful for future investigations of genes and gene networks involved in screw-worm infestations.

A large proportion of *C. hominivorax *cDNAs contain novel sequences that apparently have no significant match in any of the existing databases. This is expected, as there is very little sequence information from closely related species. Most of the non-mapped sequences are probably too divergent from sequences deposited in the available databases. A small proportion of these ESTs, however, probably derive from unique genes in *C. hominivorax*, and may include olfactory and gustatory receptors that are of interest for investigations into the feeding behavior of this parasitic species.

We used this data to investigate molecular mechanisms involved in organophosphate resistance in NWS fly. A bioassay with the dimethyl OP dichlorvos was conducted in order to select resistant individuals. Acetylcholinesterase (AChE), target of OP insecticides, is a key enzyme in the nervous system hydrolyzing excess amounts of acetylcholine into acetic acid and choline in the synapses and neuromuscular junctions [40]. None of the previously identified variants was found in the AChE sequence in any of tested groups, suggesting that OP resistance in NWS is rather due to detoxification enzymes, unless other mutations not evaluated occurred in the AChE gene. Most variants on the AChE active site might be deleterious and result in a high fitness cost. Hence, such variants are not likely to be maintained as segregating polymorphisms in the population in the absence of a strong selection pressure. As the mutated NWS carboxylesterase E3 degrades the insecticide before it can act on the AChE target site, there is no pressure to maintain the deleterious AchE variants, as verified in *L. cuprina *[18,41].

Elevated carboxylesterases levels associated with resistance to OP and carbamate insecticides have been well documented in numerous arthropod species [42]. However, altered carboxylesterase resulting in OP hydrolase activity has been described only for some dipteran species. Substitutions in NWS E3 gene were found in all individuals from the resistant and control groups. However, three different genotypes were found in control group, while all individuals resistant to dichlorvos showed a single genotype; they were all homozygous for the W251S allele. This evidence is strongly supported by biochemical studies in *L. cuprina *in which alteration in the acyl pocket of the active site also confers resistance to dimethyl OPs [20]. Although *in vitro *expression for E3 gene has not been performed in NWS, our data indicate that a similar resistance based on altered E3 has evolved in NWS fly.

Besides carboxylesterases, up-regulation of genes encoding P450 s and GSTs has been frequently associated with metabolic-based insecticide resistance mechanisms in insects [36,43]. In this context, this study is the first evaluation of the expression levels of genes putatively involved in insecticide metabolism in NWS. Of the 18 evaluated genes, only one showed differential gene expression among resistant and control groups, the putative ortholog to *CYP6G1*.

Since P450 s play a key role in detoxifying numerous xenobiotic compounds, we expected their involvement in dichlorvos metabolism. However, we did not observe an up-regulation in any of the *CYP *genes analyzed in the resistant groups (R1 and R2). The association of the high expression of the *CYP6G1 *gene with insecticide resistance is still in debate. Over-expression of the *CYP6G1 *gene was reported in *D. melanogaster *strains resistant to imidacloprid and dichlorodiphenyltrichloroethane (DDT) [44]. The over-expression in resistant individuals of *D. melanogaster *from several populations was associated with the insertion of an Accord element in the upstream regulatory region of the *CYP6G1 *gene [45,46]. The resistant individuals of *D. melanogaster *constitutively over-expressed *cyp6g1*, regardless of a previous contact with the insecticide [45,46]. In our experimental design it was not possible to distinguish the two scenarios, the constitutive low expression of *cyp6g1 *in resistant individuals or its inhibition after the insecticide treatment. The analysis of individual levels of the *cyp6g1 *transcript will shed light to this question. Contrary to these findings in *D. melanogaster*, a lack of correlation between *cyp6g1 *expression and DDT resistance was observed in other studies [47-49]. Our results add yet more complexity to this debate. A putative ortholog to *cyp6g1 *was substantially down-regulated in NWS resistant individuals and this is the first report of down-regulation of this *CYP *gene possibly associated with insecticide resistance. These intriguing *cyp6g1 *results motivate further investigations to understand the involvement of this gene in insecticide resistance.

It has been previously reported that some organophosphate insecticides require an oxidative biotransformation into more toxic structures that inhibit acetylcholinesterase, a process that is mediated by some P450 enzymes [50]. In such cases, a decrease of the expression levels of these *CYP *genes would be an advantage in the presence of an OP insecticide by preventing its bioactivation by P450 enzymes. Although this seems a reasonable explanation for our observed decrease in gene expression, it may not apply to the OP we used (dichlorvos) because its structure already contains the phosphate (P = O) biologically active as AChE inhibitors [51]. Nonetheless, the possible involvement of a reduced level of a P450 enzyme with insecticide resistance brings about a discussion on the use of synergistic compounds like PBO that inhibit P450 and esterase [52]. Our results are in line with previous studies indicating these synergists should be used with caution as in some concentrations they could reduce the insecticide efficacy [53,54].

An alternative explanation to this observation is the genetic linkage between the E3 mutant allele and the allele associated with low-expression levels of *CYP6G1*. In this case, the difference in *CYP6G1 *expression levels between the control and resistant samples may have resulted from the selection of cis- or trans-acting regulatory variants linked to the selected E3 allele. A third possible scenario for the observed changes in expression levels of *CYP6G1 *involves the alternative splicing of this gene in the presence of the insecticide [55,56]. It is possible that we measured the expression (by qRT-PCR) of exons that are skipped in the presence of the insecticide.

Contrary to our expectations, none of the 18 genes analyzed showed an increased expression level in the resistant screw-worms. Considering all P450 s, GSTs and CCEs found among in *C. hominivorax *characterized transcripts, we analyzed only some genes putatively associated with metabolic resistance and, therefore, we cannot rule out the possibility that other genes are involved in the insecticide resistance in this species. RNA sequencing is a promising approach for identifying novel genes involved in insecticide resistance and it is a natural follow-up to our analysis. Nonetheless, it is possible that the insecticide concentration we used (LC90) was too high to allow the metabolic resistance based on the over-expression of hydrolyzing enzymes. This high pressure has selected only individuals that are homozygous for the W251S allele of the E3 gene. This result, associated with the population survey [16,57], represents a strong evidence that altered carboxylesterase E3 is the major mechanism of OP insecticide resistance in NWS. Therefore, the identification of mutations in E3 gene in NWS natural populations can provide important information regarding susceptibility to some class of insecticides and contribute to implement more effective strategies for NWS control.

## Conclusions

By using 454 pyrosequencing we have sampled screwworm transcribed sequences more deeply than has been previously done, providing a large database of protein coding genes. This database is a rich resource, paving the way for future functional studies involving *C. hominivorax *and other Calliphoridae species.

We used this database to investigate the molecular base of the OP resistance in NWS fly. Absence of mutations in the target site indicates that metabolic resistance mechanisms have evolved preferentially in this species. Although no carboxylesterase E3 over-expression has been verified, high correlation between mutation W251S in this gene and resistant phenotypes strongly suggests involvement of this enzyme in dichlorvos metabolism. Real-time PCR revealed that *CYP6G1 *is notably under-expressed in resistant individuals. Further empiric studies by using RNA interference could confirm the role of these genes in the resistance phenotype. Although resistance is an inevitable consequence of intensive insecticide use, a better understanding of its genetic basis may help us to implement effective management strategies to control insect pests and reduce damage resulting from their infestations.

## Methods

### *C. hominivorax ***strains**

Screwworm larvae were collected from infested cattle wounds in Caiapônia, Goiás, Brazil. The fly culture was maintained in the laboratory for nearly one year prior to sequencing. Larvae were reared at 30°C ± 5 °C in a medium consisting of fresh ground beef supplemented with blood and water (2:1). Mature larvae were allowed to pupate in sawdust. Adults were maintained in cages (34 × 50 × 26) at 25°C and fed with a diet composed of dried milk, sugar and yeast ferment.

In order to obtain sex-specific and stage-specific transcripts, total RNA from each sample was extracted separately with Trizol (Invitrogen) from whole bodies of 20 larvae, 10 adult females and 10 adult males. Extracted RNA was treated with Terminator™ 5'-Phosphate-Dependent Exonuclease (1 unit/50 μg of total RNA) (Epicentre Biotechnologies) for producing mRNA-enriched samples by selectively digesting the ribossomal RNA. Genomic DNA contamination was removed by DNase I (Invitrogen) and the mRNA-enriched samples were further purified by using Nucleospin RNA Clean-up columns (Macherey Nagel). Quantification of RNA was performed using the fluorometer 'Qubit Quantitation Platform' (Invitrogen).

### cDNA synthesis and normalization

First-strand cDNA was generated from approximately 0.3 μg of mRNA using the SMART approach [58], according to the Trimmer cDNA normalization kit protocol (Evrogen) using the oligonucleotides CDS-3M (5'-AAG CAG TGG TAT CAA CGC AGA GTG GCC GAG GCG GCC(T)_20_VN-3') and SMART IV (5'-AAG CAG TGG TAT CAA CGC AGA GTG GCC ATT ACG GCC GGG-3'). Double-stranded cDNA was synthesized by using PCR primer IIA (5'-AAG CAG TGG TAT CAA CGC AGA G-3'). In order reduce the prevalence of abundant transcripts and increase the efficiency of rare transcript discovery, the resulting double-stranded cDNAs were normalized using a duplex-specific nuclease enzyme (Trimmer cDNA normalization kit, Evrogen) [59]. Quantification cDNA samples before each procedure was performed using the fluorometer 'Qubit Quantitation Platform' (Invitrogen).

### 454 sequencing

Approximately 5 μg of double-stranded cDNA prepared from each sample was used for library preparation following previously described methods [60]. During library preparation, sample-specific MID adaptors (Table 1) were blunt-end ligated onto each DNA fragment in a sample. The three samples were pooled prior to sequencing, which was performed on a Genome Sequencer FLX Instrument (Roche Diagnostic) following standard protocols.

### Assembly of 454-ESTs

Quality filtering and trimming of low quality read ends and of 454 adaptors were done using 454 run-time applications. SMART adaptors and MID sequences were removed from the 454 reads by using cross_match [61].

The processed reads were clustered using the MIRA v2.9.26x3 [27] assembler with the "denovo, EST, normal, 454" parameters. The default assembly options of minimum read length of 40 nucleotides, minimum sequence overlap of 40 nucleotides and a minimum relative overlap score of 80% of similarity were used.

### Analysis of library specific transcripts

To identify library-specific transcripts, raw 454-ESTs were sorted according to their MID sequence using an in-house Perl script (available on request). ESTs from each library were mapped against the assembled *C. hominivorax *unigenes using PanGEA [35] with default parameters for 454 sequences. The number of sorted reads from each library mapped to each unigene (unigene-count) was used as the representation of that unigene in the analyzed library. Unigene-counts were normalized by dividing them by the total number of reads in each library. When no read was detected in one library, a value of '1' was used to avoid any division by zero.

Fold change in representation of a given unigene was calculated by dividing the highest normalized unigene-count observed between two samples by the lowest normalized unigene-count observed between the same two samples.

### Blast searches and unigene annotation

Identity searches (tBLASTx) of unique sequences (unigenes) of *C. hominivorax *were done locally against a transcript database composed of sequences from the 12 *Drosophila *genomes (available from FlyBase [30]), *Anopheles gambiae, Aedes aegypti, Culex pipiens, Ixodes scapularis *and *Pediculus humanus *genomes (available from VectorBase [62]. The e-value cutoff was set at 1 × 10^-4 ^and only alignments with at least 50 bp were considered for unigenes with at least 50% identity with a sequence in the database over at least 50% of their length. Unigenes with no hit against this database were searched against the NCBI non-redundant 'nr' database. For the tBLASTx remote searches, we required an e-value of 1 × 10^-4 ^and a minimum of 50% of the sequence to be involved in the best hit, with at least 50% of identity. Both, local and remote, searches were automated using in-house Perl scripts (available upon request).

For gene ontology mapping, NWS unigenes were mapped to gene and transcript database of *D. melanogaster *release 5.22 (available from FlyBase). The hit with the highest E-value was considered as a putative homolog of the NWS unigene. The FlyBase identifier (FBgn#) of each *D. melanogaster *gene was used for gene ontology (GO) classification [29,31] of the mapped NWS unigenes.

### Data availability

Raw 454 reads and assembled contigs (unigenes) were submitted to the Sequence Read Archive (SRA). SFF files containing raw sequences and sequence quality information can be accessed through the SRA web site under accession number SRA020973.

### Bioassay with dichlorvos

To identify genes with an altered level of gene expression after insecticide treatment, we performed a bioassay using the OP insecticide dichlorvos (dimethyl 2,2-dichlorovinyl phosphate; C_4_H_7_Cl_2_O_4_P). Larvae from the sequenced strain-third and fourth generations-were used in two bioassays (R1 and R2) to select the resistant individuals.

Firstly, we estimated the lethal concentration for 90% of the population (LC90). Concentration-response curves were first established by exposing second instar larvae to four OP concentrations (22.5 mg/L, 11.2 mg/L, 7.4 mg/L, 5.6 mg/L). The different insecticide amounts were directly mixed to larvae medium. Log probit analysis [63] in the POLO-PC program (LeOra software) was used to calculate the LC90. Bioassays were carried out (in duplicate) by applying the estimated LC90 (20 mg/L) on 500 NWS larvae (L2 instar). Mortality was observed after 24 h exposure and larvae that survived to the treatment were collected immediately for RNA extraction.

The levels of gene expression of 18 candidate genes selected from the 454 data were analyzed in a non-treated (Control) and in the two groups (R1 and R2) that survived to dichlorvos treatment.

### Carboxylesterase and acetylcholinesterase genotyping

Based on NWS AChE previously sequenced, two sets of primers were used to amplify both regions containing three mutation sites: AChEF2 (5' CGATCCTGATCATTTAATCC 3') with AChER3 (5' TTGCAATCATTTATCAAAGC 3') and AChEF3 (5' AATCCCCAATCGGTTATG 3') with AChER2 (5' CCTCATCCTTGACATTTCC 3'), according to Silva et al [16] (Figure 7A). Direct sequencing of the PCR products was done in the ABI 377 automatic sequencer (Applied Biosystems).

PCR-RFLP was used to genotype E3 mutations (Figure 7B). To amplify the region of the E3 gene with both mutation sites forward and reverse primers (7F1aN:5' GGCTCCAGAAACTAAACG 3' and 7R3a:5' ATCCTTATCATTATTTTCACCC 3') were designed based on the E3 nucleotide sequence [4]. Endonucleases Tsp45I and Eco130I (New England Biolabs) were used to identify the G137D and W251S mutations, respectively. The digested fragments were separated by electrophoresis on 2% agarose gels and stained with ethidium bromide.

### Quantitative real-time PCR

Based on the annotation of NWS unigenes, 18 were selected for the comparison of the gene expression levels (Table 2). Primer pairs for each unigene (Additional file 5) were designed using the Primer3 software [64].

Total RNA was individually extracted from larvae that survived to both bioassays (R1 and R2) and from a subset of larvae (n = 40) of the control group (C) using Trizol (Invitrogen). Five micrograms of total RNA were treated with DNase Turbo (Ambion) and the concentration was determined fluorometrically (Qubit, Invitrogen). First-strand cDNA was synthesized using 'RevertAid™ H Minus M-MuLV Reverse Transcriptase' (Fermentas). Resulting cDNAs were diluted 40 times for PCR reactions.

Data analysis was performed according to the ΔΔ*CT *method [65] and using the gene encoding the ribosomal protein rp49 as an endogenous control on the Real-Time 7500 PCR System (Applied Biosystems). The efficiency of PCR amplification for each gene-specific primer pair was analyzed with five serial dilutions in three technical replications. The real-time PCR was carried out in 12.5 μL reactions containing 6.25 μL SYBR Green PCR Master Mix (Applied Biosystems), 0.4 μM of each primer and 4.25 μL of diluted cDNA, according to manufacturer's instructions. Thermal cycling conditions were: 95°C for 10 min, 40 cycles of 95°C for 15 s and 60°C for 60 s. For the qRT-PCRs we used three biological replicates and each reaction was performed in triplicate. Data was statistically analyzed using analysis of variance (ANOVA).

## Authors' contributions

RAC, AMLAE and TTT conceived of the study. RAC and TTT designed and carried out the experiments. TTT carried out the bioinformatic analysis, supervised the project and coordinated all activities. RAC and TTT wrote the manuscript. All authors read and approved the final manuscript.

## Supplementary Material

Additional file 1 **Insect databases used for functional annotation of *C. hominivorax *unigenes**. The table contains the source of the databases we used to annotate *C. hominivorax *unigenes as well as how many sequences were mapped to each database. Click here for file

Additional file 2 **Transcripts with different representation between the larval library and the combined adult libraries**. To calculate the fold difference in the representation of unigenes in our libraries, we used the number of reads from each library normalized by the library size (in number of reads) as a measure of the transcript abundance. Of the 645 unigenes differently represented, 356 had no read in one of the libraries and the fold change was calculated assuming a value of 1. Positive and negative values represent a highest number of reads in the adult and larva libraries, respectively. Only unigenes represented by at least 20 reads were included. Click here for file

Additional file 3 **Transcripts with different representation between male and female libraries**. To calculate the fold difference in the representation of unigenes between the two libraries, we used the number of reads from each library normalized by the library size (in number of reads) as a measure of the transcript abundance. Of the 101 unigenes differently represented, 57 was either male or female specific and the fold change was calculated assuming a value of 1 in the library with no reads. Positive and negative values represent a highest number of reads in the female and male libraries, respectively. Only unigenes represented by at least 20 reads were included. Click here for file

Additional file 4 **Gene expression levels of NWS candidate genes measured via qRT-PCR**. Averaged cycle threshold (C_T_) and Relative Quantification (RQ) for the 18 candidate genes involved in secondary metabolism. The fold difference in the gene expression was calculated between the control (C) and resistant group (R1). The only gene (*CYP6G1*) presenting a difference in level of expression more than 2-fold between these groups was re-analyzed in a biological replicate, a second resistant group (R2). Click here for file

Additional file 5 **Primer pairs used for the gene expression analysis using qRT-PCR**. Annotated NWS unigenes with a possible role in insecticide resistance were selected for the comparison of the gene expression levels. Primer pairs for each unigene were designed using the Primer3 software [64]. Click here for file
