# Supplementary material for: Deep sequencing of New World screw-worm transcripts to discover genes involved in insecticide resistance
Source: BMC Genomics. 2010 Dec 8;11:695. doi: 10.1186/1471-2164-11-695 (PMC3022914; doi:10.1186/1471-2164-11-695)
Supplement: Additional file 1 — Insect databases used for functional annotation of C. hominivorax unigenes. The table contains the source of the databases we used to annotate C. hominivorax unigenes as well as how many sequences were mapped to each database. [file 1471-2164-11-695-S1.DOC]

# Additional file 1 for “Deep sequencing of New World screw-worm transcripts to discover genes involved in insecticide resistance”

### Renato Assis de Carvalho, Ana Maria Lima de Azeredo-Espin, Tatiana Teixeira Torres

tttorres@unicamp.br

## *Supplementary table 1 - Insect databases used for functional annotation of C. hominivorax unigenes*

| **Species** | **Source** | **Release** | **Number of unigenes mapped** |
| --- | --- | --- | --- |
| *Aedes aegypti* | Vectorbase.org | AaegL1.1 | 320 |
| *Anopheles gambiae* | Vectorbase.org | AgamP3.5 | 265 |
| *Culex pipiens* | Vectorbase.org | CpipJ1.2 | 636 |
| *Drosophila annanassae* | Flybase.org | 1.3 | 1446 |
| *Drosophila erecta* | Flybase.org | 1.3 | 411 |
| *Drosophila grimshawi* | Flybase.org | 1.3 | 1338 |
| *Drosophila melanogaster* | Flybase.org | 5.22 | 1413 |
| *Drosophila mojavensis* | Flybase.org | 1.3 | 1814 |
| *Drosophila persimilis* | Flybase.org | 1.3 | 332 |
| *Drosophila pseudoobscura* | Flybase.org | 2.6 | 1390 |
| *Drosophila sechellia* | Flybase.org | 1.3 | 293 |
| *Drosophila simulans* | Flybase.org | 1.3 | 419 |
| *Drosophila virilis* | Flybase.org | 1.2 | 2103 |
| *Drosophila willistoni* | Flybase.org | 1.3 | 2542 |
| *Drosophila yakuba* | Flybase.org | 1.3 | 1481 |
| *Ixodes scapularis* | Vectorbase.org | IscaW1.1 | 52 |
| *Pediculus humanus* | Vectorbase.org | PhumU1.1 | 104 |
