# Supplementary material for: Deep sequencing of New World screw-worm transcripts to discover genes involved in insecticide resistance
Source: BMC Genomics. 2010 Dec 8;11:695. doi: 10.1186/1471-2164-11-695 (PMC3022914; doi:10.1186/1471-2164-11-695)
Supplement: Additional file 4 — Gene expression levels of NWS candidate genes measured via qRT-PCR. Averaged cycle threshold (CT) and Relative Quantification (RQ) for the 18 candidate genes involved in secondary metabolism. The fold difference in the gene expression was calculated between the control (C) and resistant group (R1). The only gene (CYP6G1) presenting a difference in level of expression more than 2-fold between these groups was re-analyzed in a biological replicate, a second resistant group (R2). [file 1471-2164-11-695-S4.DOC]

# Additional file 4 for “Deep sequencing of New World screw-worm transcripts to discover genes involved in insecticide resistance”

### Renato Assis de Carvalho, Ana Maria Lima de Azeredo-Espin, Tatiana Teixeira Torres

tttorres@ib.usp.br

### Supplementary table 4 - Gene expression levels of NWS candidate genes measured via qRT-PCR

| **Gene family** | **Gene** | **AvC_T_**  **(E)** | **AvC_T_**  **(C)** | **AvC_T_**  **(R)** | **ΔC_T_** | **ΔΔC_T_** | **RQ**  **(2^-ddCt^)** |
| --- | --- | --- | --- | --- | --- | --- | --- |
| Esterases | Acetylcholinesterase | 18.246 | 29.889 | 29.965 | 11.718 | 0.707 | 0.612 |
|  | Alpha-Esterase-7 (*EST7*) | 16.894 | 24.799 | 24.251 | 7.357 | -0.846 | 1.797 |
|  | Alpha-Esterase-8 (*EST8*) | 16.894 | 25.736 | 26.884 | 9.990 | 0.850 | 0.554 |
|  | Alpha-Esterase-9 (*EST9*) | 16.894 | 26.283 | 27.348 | 10.454 | 0.767 | 0.587 |
|  | Serineprotease 7 | 16.894 | 21.883 | 22.158 | 5.264 | -0.022 | 1.015 |
| GST | GlutathioneS transferase D1 | 16.894 | 21.941 | 22.436 | 5.542 | 0.197 | 0.872 |
|  | GlutathioneS transferase E5 | 16.894 | 17.605 | 18.792 | 1.898 | 0.888 | 0.540 |
|  | GlutathioneS transferase S1 | 16.894 | 21.016 | 20.861 | 3.967 | -0.453 | 1.369 |
| P450 | Cyp4ac1 | 16.894 | 28.073 | 27.159 | 10.265 | -0.879 | 1.839 |
|  | Cyp4c3 | 16.894 | 26.445 | 27.641 | 10.747 | 0.898 | 0.536 |
|  | Cyp4d2 | 16.894 | 26.487 | 27.744 | 10.850 | 0.958 | 0.514 |
|  | Cyp6a14 | 16.894 | 23.273 | 23.410 | 6.516 | -0.161 | 1.118 |
|  | Cyp6a9 | 16.894 | 26.104 | 26.811 | 9.917 | 0.408 | 0.753 |
|  | Cyp6d4 | 17.453 | 24.304 | 25.083 | 7.629 | 0.944 | 0.519 |
|  | Cyp6g1 | 16.382 | 17.028 | 22.346 | 5.964 | 5.510 | **0.021** |
|  | Cyp6g1 (R2) | 15.503 | 17.028 | 19.263 | 3.760 | 3.306 | **0.101** |
|  | Cyp6v1 | 17.453 | 27.552 | 26.682 | 9.229 | -0.703 | 1.628 |
|  | Cyp9f2 | 17.453 | 21.521 | 21.660 | 4.206 | 0.304 | 0.809 |
|  | Cyp12a4 | 17.453 | 24.353 | 24.830 | 7.377 | 0.643 | 0.640 |

E, endogenous control, *rp49*

C, larvae not treated with insecticide

R, larvae resistant to insecticide

RQ, relative quantification (resistant, R to control, C)
