# Supplementary material for: Deep sequencing of New World screw-worm transcripts to discover genes involved in insecticide resistance
Source: BMC Genomics. 2010 Dec 8;11:695. doi: 10.1186/1471-2164-11-695 (PMC3022914; doi:10.1186/1471-2164-11-695)
Supplement: Additional file 5 — Primer pairs used for the gene expression analysis using qRT-PCR. Annotated NWS unigenes with a possible role in insecticide resistance were selected for the comparison of the gene expression levels. Primer pairs for each unigene were designed using the Primer3 software [64]. [file 1471-2164-11-695-S5.DOC]

# Additional file 5 for “Deep sequencing of New World screw-worm transcripts to discover genes involved in insecticide resistance”

### Renato Assis de Carvalho, Ana Maria Lima de Azeredo-Espin, Tatiana Teixeira Torres

tttorres@ib.usp.br

### Supplementary table 5 - Primer pairs used for the gene expression analysis using qRT-PCR

| **Gene** | **Primer sequence (5’ → 3’)** | **T_a_ (^o^C)** | **Product size (bp)** |
| --- | --- | --- | --- |
| Ribosomal protein L32 (rp49) | F: GCACCAAGCACTTCATCC  R: AGTGGGAAGCATGTGACG | 60 | 169 |
| Acetylcholinesterase | F: TGCAATTGTAATGCCTCG  R: GGAATTCCATTGTTGTACCG | 60 | 102 |
| Alpha-Esterase-7 (*EST7*) | F: CAACACCTTGGGATGG  R: CTGGACGTTTAGTTTCTGG | 60 | 150 |
| Alpha-Esterase-8 (*EST8*) | F: CGCACAGCCATTTAATGAAG  R: ATGTGGTCGCAAAGTACGTG | 60 | 208 |
| Alpha-Esterase-9 (*EST9*) | F: GGACCGAAGCCTTTACAGAA  R: GCCAAACTGAAAACCACCAC | 60 | 150 |
| Serineprotease 7 | F: TGGAGCTAAACAACACCAACAC  R: CGGTGTCATTGCCATTGTAG | 60 | 135 |
| GlutathioneS transferase D1 | F: AAAATCAACCTGCCGATCC  R: GCCAATAAAGCTAAATCGGCTA | 60 | 130 |
| GlutathioneS transferase E5 | F: TACGTAAACCGCCTTGGAAC  R: TGCCATCATTGCTTACTTGG | 60 | 134 |
| GlutathioneS transferase S1 | F: GCAGTCGTCTCCTACGAACC  R: TTCAATGCCAAATGACCATC | 60 | 134 |
| Cyp4ac1 | F: ATTGCATTGGCCAAAAATTC  R: AATCTTCCAATTTTGTTATGGGTA | 60 | 97 |
| Cyp4c3 | F: CCGGTTATTCTCCCATAACG  R: GATGGCATTACCCAAGAAAG | 60 | 143 |
| Cyp4d2 | F: TTTGCTGTTATGCGTGATCC  R: CCAGCCGAAAAAGGAACATA | 60 | 119 |
| Cyp6a14 | F: ATGCCATTGTGGTAGAGGAAG  R: AGATCTTTCCCAGGGTTTGAC | 60 | 95 |
| Cyp6a9 | F: AGCAAAACCCATGGTAGAGG  R: CAAAACCGAACATGGTGATG | 60 | 115 |
| Cyp6d4 | F: TGAAGGGTATGTTTGGTACGG  R: AACCAAAAATTGCCGAAGC | 60 | 156 |
| Cyp6g1 | F: CGGTTTCTGCTTCTTTACGG  R: CTTCTCTCCCGAAACCACTG | 60 | 131 |
| Cyp6v1 | F: CCAACCGACCCATTTATTG  R: ATAAAGCCGGTTGATGGAAC | 60 | 146 |
| Cyp9f2 | F: TCAATGTTTCCTATTCTTCTTTGC  R: CTTGACATTCGGGATTTTCC | 60 | 95 |
| Cyp12a4 | F: AAAGCATTATCCGTGGGATG  R: ATGTCCTCATGCCCTCAAAC | 60 | 168 |
